# Self-Reported Memory Problems Are Related to Depressive Symptoms in Older Adults With Certain Personality Traits

**DOI:** 10.1093/geroni/igaa057.1254

**Published:** 2020-12-16

**Authors:** Sakshi Bhargava, Nikki Hill, Jacqueline Mogle

**Affiliations:** 1 The Pennsylvania State University, University Park, Pennsylvania, United States; 2 Pennsylvania State University, University Park, Pennsylvania, United States

## Abstract

Self-reported memory problems and depressive symptoms tend to co-occur in older adults; however, this relationship may depend on personality traits and the type of self-reported memory assessed. Using a coordinated analytic framework, this study examined whether neuroticism, extraversion, and conscientiousness moderated the associations of older adults’ self-reported memory with depressive symptoms at between- and within-person levels across three large, longitudinal datasets (range=8-12 years of follow-up) of community-dwelling older adults with no evidence of cognitive impairment (n=427-6,960; Mage: 69.47- 75.94; 72-84% White; 60-64% Female). Assessments of depressive symptoms (GDS-15 or CES-D) and self-reported memory (perceived memory decline, frequency of forgetting, and current memory rating) were taken annually or biennially; personality was assessed via the IPIP or NEO Five-Factor Inventory. Results were largely consistent across datasets. Specifically, between persons, self-reported memory problems (including perceived memory decline, higher frequency of forgetting, and lower current memory rating) were related to higher depressive symptoms only among older adults higher in neuroticism. In one dataset, results supported a protective effect of conscientiousness such that higher frequency of forgetting was related to lower depressive symptoms among older adults higher in this trait. Within persons and across datasets, at times when perceived memory decline was reported, or current memory rating was lower, depressive symptoms tended to be higher only in older adults higher in neuroticism. Results demonstrate the importance of considering personality traits and the type of self-reported memory when examining associations among reports of memory problems and depressive symptoms in cognitively intact older adults.

